# Acupuncture Versus Standard Medical Care in the Prophylactic Treatment of Migraine: A Systematic Review and Meta‐Analysis

**DOI:** 10.1111/ene.70160

**Published:** 2025-04-16

**Authors:** Francesca Pistoia, Simone Cesarano, Gennaro Saporito, Maria Albanese, Cecilia Lucenti, Secondo Scarsella, Aldo Liguori, Federica Umani Ronchi

**Affiliations:** ^1^ Department of Biotechnological and Applied Clinical Sciences University of L'aquila L'Aquila Italy; ^2^ Regional Referral Headache Center San Salvatore Hospital L'Aquila Italy; ^3^ Department of System Medicine University of Rome Tor Vergata Rome Italy; ^4^ Integrated Medical Acupuncture Siena Italy; ^5^ Maxillofacial Surgery San Salvatore Hospital L'Aquila Italy; ^6^ Paracelso Institute Rome Italy; ^7^ Department of Anatomical, Histological, Forensic and Orthopedic Sciences Sapienza University Rome Italy

**Keywords:** acupuncture, migraine, prophylaxis, quality of life

## Abstract

**Background:**

Although innovative pharmacological therapies for migraine prevention are now available, they may not be suitable or effective for all patients due to concerns about tolerability and the varying complexity of the underlying condition. This study systematically reviewed and meta‐analyzed acupuncture's effects on migraine prophylaxis compared to standard medical care, focusing on study heterogeneity and issues related to sham interventions.

**Methods:**

Following the PRISMA guidelines and using the PICO model, the study searched PubMed, Scopus, CNKI, and VIP database from December 1965 to September 2024. Studies evaluating acupuncture's clinical efficacy for migraine prophylaxis, including clinical trials, observational studies, case series, and case reports, were considered. An additional search was conducted on the clinicaltrials.gov database from the beginning of indexing up to September 2024 to include ongoing studies. Quality control and bias assessment were performed. Primary outcomes focused on acupuncture's efficacy and safety versus pharmacological treatments in reducing migraine frequency and intensity. The impact on patients' quality of life was also evaluated.

**Results:**

At the end of the selection process, 15 studies were eligible. Acupuncture showed no statistically significant difference as a prophylactic treatment for migraine in reducing the frequency of migraine days or pain intensity but did reduce the use of analgesics while improving patients' quality of life.

**Conclusion:**

Current evidence supports acupuncture as an adjunctive therapy in migraine prophylaxis, but challenges such as protocol heterogeneity, dropout biases, the complexities of sham‐controlled trials, and the lack of comparison data with newer innovative treatments not yet considered warrant further research.

## 1 Introduction

Migraine is a widespread condition that causes significant disability in affected individuals and considerable costs on society due to patient management and lost productivity [1, 2]. Compared to the past, migraine prophylaxis is much more effective today, thanks to the use of traditional medications (beta‐blockers, tricyclic antidepressants, antiepileptics, and onabotulinumtoxinA) as well as newer prophylactic treatments such as monoclonal antibodies (erenumab, galcanezumab, fremanezumab, eptinezumab) and gepants (rimegepant, atogepant, and ubrogepant). However, not all patients respond to these treatments in the same way: there are patients with an optimal response, known as super‐responders, patients who respond later, called late responders, and patients who do not respond at all, considered refractory. The most challenging patients to treat are those with chronic pain, central sensitization, a long disease duration, and allodynia [3]. In these patients, pharmacological treatment alone may not be sufficient to improve the disease course and the quality of life. Additionally, there are patients, such as adolescents or pregnant women, for whom the use of these medications is not recommended, as comprehensive safety data are not yet available [3]. Hence, the usefulness of other therapeutic approaches, such as acupuncture and auriculotherapy, which can serve as add‐on or complementary treatments. However, the efficacy of these treatments has not been proven unequivocally, due to the heterogeneity of research protocols used as well as the difficulties in designing sham trials.

The aim of this study was to conduct a systematic review and meta‐analysis on the effects of acupuncture for migraine prophylaxis compared to standard medical care, with particular attention to heterogeneity and sham‐related issues.

## 2 Materials and Methods

### 2.1 Registration, Data Sources, Searches, and Study Selection

This systematic review was performed according to the Preferred Reporting Items for Systematic Reviews and Meta‐Analyses (PRISMA) guidelines, updated to 2020 [4]. The study was registered in PROSPERO (ID: CRD42024590991), and the protocol can be found at the PROSPERO database. No ethical board approval or written informed consent from patients was necessary because of the study's design. The PICO model was used to develop the search strategy: a systematic literature search was performed to identify available randomized controlled trials (RCTs) including patients with migraine (P: population) eligible to receive acupuncture or auriculotherapy (I: intervention) vs. standard preventive pharmacological treatment (C: comparator). Reporting of functional outcomes during follow‐up (O: outcome) was a requirement for studies to be eligible for inclusion [5]. The search was conducted by identifying articles indexed from December 1965 to September 2024 on PubMed, Scopus, Chinese National Knowledge Infrastructure (CNKI), and Chinese Science and Technology.

Periodical Database (VIP). The search strategies was based on a combination of subject headings (e.g., Medical Subject Headings [MeSH] in PubMed) and the following terms: “migraine”, “acupuncture”, “electroacupuncture”, “acupressure”, “auriculotherapy” and “calcium channel blockers”, “anticonvulsants”, “beta adrenergic blocking” or tricyclic antidepressive or “botulinum toxins” or “calcitonin gene related peptide”. The search was restricted to humans, only studies focusing on the application of acupuncture in terms of clinical efficacy in the prophylactic treatment of migraine have been considered. Specifically, original studies, including clinical trials, observational studies, case series, and case reports addressing acupuncture for the treatment of migraine were eligible for this review. Studies lacking a clear definition of the study design and settings, letters, abstracts, studies not performed on humans, unpublished studies, and studies in which acupuncture was used for purposes other than the treatment of migraine were excluded. Duplicate publications were removed by manual check. The study selection process occurred through two phases: in the first phase, studies were selected through the reading of titles/abstracts, and in the second phase, through the reading of full texts. All articles were imported into an online software (www.rayyan.ai) used during the screening process. The article selection process was carried out independently by two investigators (SC and GS) who initially assessed the study eligibility by screening titles and abstracts. Disagreements were resolved through discussion with a third investigator (FP). In the second phase, the full texts of the selected articles were evaluated. To also include ongoing studies, we conducted an additional search on the clinicaltrials.gov database from the beginning of indexing up to September 2024, using the same terms as those used for the database search.

### 2.2 Quality Control and Bias Assessment

Eligible studies quality control and bias assessment were independently performed by two reviewers (SC and GS) using the Cochrane Collaboration Risk of Bias (RoB 2) tool for RCTs [6]. Disagreements were resolved through discussion and consensus with the corresponding author (FP). For data extraction, structured forms were used to record trial names, year of study execution, patient samples, characteristics, and outcomes of interest.

### 2.3 Outcomes

Main investigated outcomes were the efficacy and safety of acupuncture compared to pharmacological treatments in reducing the frequency and intensity of migraine episodes both at the end of the treatment period and during subsequent follow‐ups. Additionally, the study evaluated the impact on patients' quality of life. Data on the various outcomes were collected at the longest available follow‐up in each study. Only studies that provided clear outcome data in terms of mean and standard deviation were included in the analysis. Therefore, studies reporting outcomes with median values, ranges, or as overall percentages of the sample were excluded.

### 2.4 Statistical Analysis

Continuous outcomes were evaluated by calculating the mean difference (MD) for each study, with the associated uncertainty presented as a 95% confidence interval (CI) when the same tool was employed across studies to measure the outcome. When different tools were used, the standardized mean difference (SMD) was applied instead; the SMD value was interpreted based on the classification by Cohen et al. [7] To account for heterogeneity among studies, arising from differences in treatment protocols, assessment methods, risk of bias, and other factors that could influence the direction of the treatment effect, a random‐effects model was applied to compare the data for each outcome. The assessment of the heterogeneity was performed with the *Q*‐test, with a significant threshold of alpha = 0.1, while inconsistency among studies was quantified by I2 statistics [8]. We considered values < 25% as low heterogeneity, between 25% and 50% moderate heterogeneity, and > 50% significant heterogeneity. Prediction intervals were also calculated for both the outcomes of interest. Publication biases were assessed using both funnel plot inspection and Egger linear regression test, considering statistically significant a two‐sided *p*‐value < 0.1 [9].

## 3 Results

### 3.1 Literature Search and Included Studies

**FIGURE 1:**
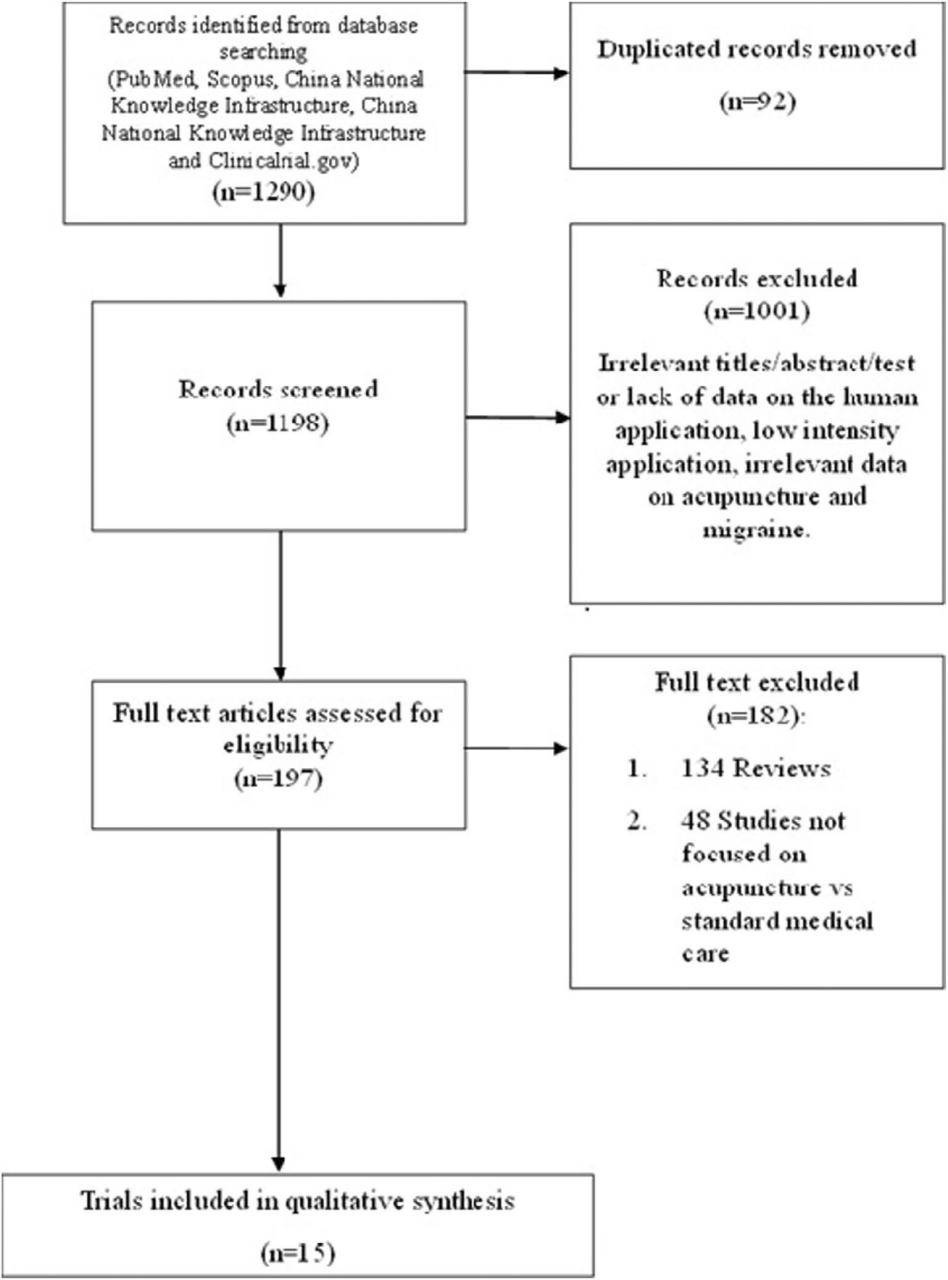
Flow diagram of selected studies.

A total of 1290 records were identified through the search on PubMed, Scopus, CNKI, and VIP. After removing duplicates, 1198 articles underwent screening, with 197 considered relevant for full‐text analysis. One hundred eighty‐two studies were subsequently excluded: 134 did not meet the eligibility criteria in terms of study design and 48 did not focus on comparison between acupuncture and medical treatment. Regarding ongoing studies, a search on clinicaltrial.gov retrieved 26 articles, but none were considered eligible for this review. At the end of the selection process, 15 studies were considered eligible (Table 1). The PRISMA flow chart is shown in Figure 1.

**TABLE 1:** Characteristics of included studies.

Study	Design, country	Population	Disease	Type and target of acupuncture	Outcomes and assessment time points	Results	Side effects
Allais et al. (2002)	Prospective, randomized, controlled trial, Italy	Acupuncture group: *n* = 80, mean age 38.4 ± 9.7 years, age at headache onset 17.9 ± 9.1 years, monthly attacks 6.4 ± 5.8, days with analgesic intake 9.7 ± 10.9 Flunarizine group: *n* = 80, mean age 37.2 ± 9.3 years, age at headache onset 17.6 ± 8.8 years, monthly attacks 6.1 ± 5.3, days with analgesic intake 9.5 ± 11.2	Migraine without aura	– LR3 Taichong – SP6 Sanyinjiao – ST36 Zusanli – CV12 Zhong‐ wan – LI4 Hegu – PC6 Neiguan – GB20 Fengchi – GB14 Yangbai – EX‐HN5 Taiyang – GV20 Baihui Bilateral application	– Number of attacks per month – Pain intensity assessed using a four‐level semantic and behavioral scale – Number of headache rescue medications – T1: 2 months – T2: 4 months – T3: 6 months	Significant difference in terms of number of attacks (*p* < 0.01), and drug intake (*p* < 0.05) in both groups. Pain intensity reduced in the acupuncture group (*p* < 0.05)	Acupuncture group: sedation following treatment (10%) and local pain (8%). Flunarizine group: drowsiness (35%), weight gain (22%), and depression (7%)
Diener et al. (2006)	Prospective, randomized, double‐blinded, control trial, Germany	Acupuncture group: *n* = 290, 85% women, 52% migraine with aura, mean age 37.1 ± 10.5 years, disease duration 201.6 ± 150.9 months, monthly attacks 3.8 ± 3.0 Sham acupuncture group: *n* = 317, 81% women, 52% migraine without aura, mean age 38.3 ± 10.4 years, disease duration 199.3 ± 131.7 months, monthly attacks 3.8 ± 3.0 Standard treatment group: *n* = 187, 82% women, 53% migraine with aura, mean age 36.8 ± 10.4 years, disease duration 184.7 ± 132.5 months, monthly attacks 4.2 ± 2.6	Migraine with or without aura	Not specified	– Difference in migraine days – Changes in pain intensity, pain‐related impairment and in quality of life – T1: 8 weeks – T2: 23–26 weeks after randomization	Mean reduction of 2.3 days in the acupuncture group (95% CI 1.9–2.7), 1.5 days (95% CI 1.1–2.0) in the sham group, and 2.1 days (1.5–2.7) in the standard treatment group. This difference was significant for all three groups (*p* < 0.001) No significant differences between the three treatment groups (*p* = 0.09). With respect to secondary outcomes, no differences between treatment groups	Nervous system side effects acupuncture group: 17 (5%); sham group: 23 (7%); standard treatment group 15 (8%) or musculoskeletal side effects acupuncture group: 19 (6%); sham group: 22 (7%); standard treatment group: 8 (4%). Overall pattern of side effects similar across all groups
Facco et al. (2013)	Prospective, randomized, controlled trial, Italy	Acupuncture group: *n* = 50, median (IQR) age 34 (32–36) years, median (IQR) Migraine Disability Assessment [MIDAS] score 44 (20–27), median (IQR) pain intensity 7 (6–8) Valproic acid group: *n* = 50, median (IQR) age 40 (36–44) years, median (IQR) MIDAS score 24 (21–26), median (IQR) pain intensity 7 (6–8)	Migraine without aura	Standard, – GB20 – St8 – EX‐HN5 – GB8 – BL12 – BL60 – TE5 – GV14 – St40 – SP6 – CV12	– MIDAS score – Pain relief score (PRS) – VAS score – Rizatriptan intake – T1: 3 months – T2: 6 months	MIDAS score reduction similar between the two groups VAS and PRS: Valproic acid group: better improvement at T1 (*p* < 0.0001), while acupuncture group greater improvement at T2 (*p* = 0.02) Rizatriptan: Valproic acid group significant increase, while acupuncture group significant decrease from T1 to T2 (*p* = 0.001)	Reported only for Valproic acid group: nausea (5 cases), constipation (4 cases), abdominal pain (5 cases), drowsiness (3 cases), weight gain (2 cases) and itching (1 case)
Giannini et al. (2021)	Prospective, randomized, sham‐controlled trial, Italy	Acupuncture group: *n* = 69, mean age 33.6 ± 17.4 years, mean age at headache onset 16.2 ± 8.6 years, mean disease duration 24.1 ± 9.9 years, monthly attacks 5.8 ± 2.2, monthly headache days 8.4 ± 2.9, monthly days with analgesic intake 8.2 ± 4.5, median (IQR) MIDAS score 21 (10–44) Standard oral therapy *n* = 66, mean age 34.7 ± 16.5 years, mean age at headache onset 15.4 ± 8.9 years, mean disease duration 24.3 ± 9.8 years, monthly attacks 5.8 ± 2.1, monthly headache days 8.3 ± 2.7, monthly days with analgesic intake 8.3 ± 4.3, median (IQR) MIDAS score 22 (8.5–44.5) Drug distribution: amitriptyline (*n* = 17; 25.8%), beta‐blockers (*n* = 7; 10.6%), flunarizine (*n* = 14;21.2%), flunarizine+riboflavin (*n* = 1;1.5%), (vitamin B2), topiramate (*n* = 9; 13.6%), pizotifen (*n* = 3;4.5%), valproic acid (*n* = 2;3.0%), duloxetine + coenzyme Q10 (*n* = 1;1.5%), riboflavin (*n* = 11;16.7%) and a combination of other nutraceutical drugs (2;3.0%)	Migraine with or without aura	– LR 3 – GB 34 – SP6 – LI 4 – TE 5 – GV20 – ST 8 – BL2 – GB 4 – GB 8 – GB 20 – BL12	– Frequency of the migraine – T1: 1 month – T2: 4 months	Migraine days significantly reduced in both groups (*p* < 0.001): from 8.58 ± 3.21 to 6.43 ± 3.45 in acupuncture group and from 8.29 ± 2.72 to 6.27 ± 4.01 in standard oral therapy group	No serious adverse events in the acupuncture group. Two adverse events requiring the suspension of the treatment (depression and hypertransaminasemia respectively following the introduction of flunarizine and topiramate)
Hesse et al. (1994)	Prospective, randomized, controlled trial, Denmark	Acupuncture treatment plus placebo tablets group *n* = 38, mean age 42.9 years minimum 26 maximum 66, 87% females, migraine duration 20.3 years minimum 2 maximum 40 Metoprolol plus sham stimulation group *n* = 39, mean age 46.5 years minimum 25 maximum 70, 82% females, migraine duration 26.5 years minimum 2 maximum 55	Migraine with or without aura	Acupoints selected individually by the therapist based on the patient's clinical response	– Frequency of migraine attacks – Global rating of attacks severity End of treatment, 17 weeks	No significant differences between treatments in attack frequency (*p* > 0.20) Significant difference in the global rating of attacks in favor of metoprolol (*p* < 0.05)	Number of patients with side effects higher in the propranolol group (*n* = 14; 36%, mainly represented by fatigue, gastrointestinal symptoms and dizziness) as compared to the acupuncture group (*n* = 3; 8%, mainly represented by gastrointestinal symptoms)
Liu et al. (2024)	Prospective randomized, controlled trial, China	Acupuncture plus placebo tablets group: *n* = 30, mean age 45.4 ± 11.9 years, 83.3% females, migraine duration 17.7 ± 9.1 years Topiramate plus sham stimulation group: *n* = 30, mean age 46.1 ± 10.7 years, 80% females, migraine duration 17.9 ± 8.6	Chronic migraine with or without aura	– GV20 – GV24 – Bilateral GB13 – Bilateral GB8 – Bilateral GB20 – TE5 – GB34 – LI4 – ST44 – BL60 – SI3 – LR3 – GB40	– Mean change from baseline in monthly migraine days (MMDs) – Disability scores (HIT 6 and MIDAS) – Quality of life scores (MSQOL) – T1: 12 weeks – T2: weeks 13–24	Significantly greater ≥ 50% reduction in MMDs in the acupuncture group as compared to topiramate group during both weeks 1–12 (33.3% vs. 6.7%; OR 9.17 [95% CI: 1.72–48.85]; *p* = 0.009) and weeks 13–24 (36.7% vs. 10.0%; OR 7.00 [95% CI: 1.61–30.46]; *p* = 0.009) Higher reductions in disability scores (HIT 6 and MIDAS) and greater improvement in quality‐of‐life scores (MSQOL) in the acupuncture group	Fewer adverse events in the acupuncture group (10%) as compared to the topiramate group (26.7%, *p* < 0.001)
Naderinabi et al. (2017)	Prospective randomized, controlled trial, Iran	Acupuncture group: *n* = 50, mean age 37.2 ± 7.3 years, disease duration 10.34 ± 5.46 years, monthly headache days 21.26 ± 6.84, days with analgesic intake 14.56 ± 5.62, Visual Analog Scale score 8.56 ± 1.29 Botulinum toxin A group: *n* = 50, mean age 36.8 ± 7.4 years, disease duration 9.22 ± 5.31 years, monthly headache days 23.56 ± 6.46, days with analgesic intake 17.76 ± 6.18, Visual Analog Scale score 8.9 ± 1.24 Oral valproate group: *n* = 50, mean age 37.6 ± 7.4 years, disease duration 9.15 ± 3.95 years, monthly headache days 21.02 ± 4.36, days with analgesic intake 14.1 ± 5.06, Visual Analog Scale score 8.36 ± 1.39	Chronic migraine	– GB 41 – GB 20 – GB 15 – GB14 – GB10 – GB8, – LI 4 – Liver 3 – Sanjiao 5 – Du‐Mai 20	– MMDs – VAS – MMDs with the need of medications – Patients missing workdays (%) due to headache – T1: 1 month – T2: 2 months – T3: 3 months	MMDs significantly reduced from baseline to T3 in all the groups (*p* = 0.0001); greater reduction in the acupuncture group both at T1 (*p* = 0.0001) T2 (*p* = 0.0001), and T3 (*p* = 0.0001) VAS significantly reduced from baseline to T3 in all the groups (*p* = 0.001); greater reduction in the acupuncture group both at T1 (*p* = 0.0001), T2 (*p* = 0.001) and T3 (*p* = 0.0001) MMDs with the need of medications significantly reduced in all the groups (*p* = 0.001); greater reduction in the acupuncture group both at T1 (*p* = 0.0001), T2 (*p* = 0.001) and T3 (*p* = 0.0001) Proportion of patients missing workdays (%) due to headache significantly reduced across all groups at all study times (*p* = 0.0001). No significant differences between groups at T0 (*p* = 0.443), T2 (*p* = 0.827), and T3 (*p* = 0.179). Greater reduction in the botulinum toxin A group at T1 (*p* = 0.023)	Significantly less side effects in the acupuncture group (*p* = 0.021). Acupuncture side effects; bleeding or subcutaneous hematoma Botulinum toxin A side effects: ptosis, facial masking, or asymmetry Sodium valproate side effects: asthenia, anorexia, weight gain, tremor, somnolence, insomnia, and alopecia
Quiarnu et al. (2020)	Prospective, randomized, controlled trial, China	Acupuncture group: *n* = 22, mean age 39.16 ± 2.31, migraine duration 8.45 ± 1.17 Flunarizine plus gabapentin group: *n* = 23, mean age 38.46 ± 1.24 years, migraine duration 7.56 ± 2.17 years	Migraine with or without aura	– EX‐HN5 – GB20 – ST8 – GB8 – LR2 – LR3 – SP9 – ST40 – GB39 – SJ5 – LI11 – BL17 – SP6 – SP10 – KI3	– VAS – Frequency of migraine attacks – Pain duration (h/die) – T1: 4 weeks – T2: 12 weeks	VAS: significantly reduced in both group at T1 e T2 (Acupuncture group baseline: 5.74 ± 0.09; T1: 5.38 ± 1.35; T2: 3.84 ± 0.87; Flunarizine group baseline: 5.73 ± 0.07; T1: 4.41 ± 1.15; T2: 2.76 ± 0.65). greater reduction in the Flunarizine group *p* < 0.05. frequency and severity of the attacks are significantly reduced in both groups, with a significantly greater reduction in the Flunarizine group, *p* < 0.05	Flunarizine group showed mild and transient adverse events: dizziness, nausea, and drowsiness. Meanwhile, the acupuncture group showed no adverse effects
Streng et al. (2006)	Prospective, randomized, controlled trial, Germany	Acupuncture group: *n* = 59, mean age 40.0 ± 11.4 years, migraine duration 14.5 ± 9.9 years, monthly headache days 8.0 ± 3.4, monthly migraine days 3.0 ± 1.4 Metoprolol group: *n* = 55, mean age 40.3 ± 10.7 years, migraine duration 17.3 ± 10.8 years, monthly headache days 7.3 ± 2.7, monthly migraine days 2.9 ± 1.3.	Migraine with or without aura	– Gall Bladder 20, 40 or 41 or 42 – Du Mai 20 – Liver 3 – San Jiao 3 or 5	– MMDs – VAS – Disability scores (Physical health SF‐36 and Mental health SF‐36) – T1: 9 weeks – T2: 12 weeks	MMDs reduction by 2.5 ± 2.9 days in the acupuncture group compared to 2.2 ± 2.7 days in the metoprolol group (difference acupuncture vs. metoprolol 0.2 days, 95% CI −1.0 to 1.5 days, *p* = 0.721; 2‐sided exploratory testing, intent‐to‐treat population) – VAS reduction in both groups (Acupuncture vs. Metoprolol: −6.5 [−11.4 to −1.6], *p* = 0.032) – SF‐36 Physical Health domain reduction in both groups (Acupuncture vs. Metoprolol: 3.8 [0.3–7.3], *p* = 0.034)	No serious adverse in the acupuncture group; severe side effects in the metoprolol group in six patients. Higher drop‐out rate in metoprolol group
Wang et al. (2011)	Prospective, randomized, controlled trial, China	Acupuncture plus placebo tablets group: *n* = 70, mean age 39.2 ± 10.9 years, 84.3% females, migraine monthly days 7.1 ± 3.5 days, mean ± SD VAS 6.9 ± 1.7 Flunarizine plus sham stimulation group: *n* = 70, mean age 39.9 ± 13.1 years, 85.7% females, migraine monthly days 6.2 ± 3.4 days, mean ± SD VAS 6.7 ± 1.9	Migraine without aura	Standard, – DU20 – DU24 – GB13 – GB8 – GB20 – SJ5 – GB34 – LI 4 – ST 44 – BL 60 – SI 3 – LR3 – GB40 – PC6 – LR3	– Proportion of patients with a reduction of MMDs by at least 50% – MMDs – VAS – 36‐item short‐form health survey (SF‐36) physical and mental component improvement – T1: 4 weeks – T2: 16 weeks	Better response rates (41% vs. 28% at week 4 and 39% vs. 26% at week 16, *p* < 0.05) and a greater reduction in MMDs (difference from baseline in 4.1 ± 3.5 vs. 1.9 ± 2.3 at week 4 and 4.1 ± 3.5 vs. 2.0 ± 2.7 at week 16, *p* < 0.05) in the acupuncture group as compared to the control group No significant differences between groups in VAS scores and SF‐ 36 physical and mental component summary scores (*p* > 0.05)	Mild adverse effects in 5 patients in the acupuncture group (3 cases of mild bleeding after needle removal without formation of subcutaneous hematoma, 1 case of scalp discomfort and 1 case of fatigue with unknown cause) and in 7 patients in the control group (5 cases of fatigue or faintness and 2 cases of weight gain)
Xu et al. (2020)	Prospective, randomized, controlled trial, China	Acupuncture plus usual treatment group: *n* = 60, mean age 36.6 ± 12.0 years, 78% females, migraine monthly days 5.8 ± 2.6 days, mean ± SD VAS 5.1 ± 1.3 Sham acupuncture plus usual treatment group: *n* = 60, mean age 36.0 ± 10.9 years, 83% females, MMD 6.3 ± 3.8 days, mean ± SD VAS 5.3 ± 1.3 Usual treatment alone *n* = 30, mean age 37.3 ± 11.7 years, 87% females, MMD 5.8 ± 3.0 days, mean ± SD VAS 5.1 ± 1.8	Migraine without aura	– L14 – LR3 – EX‐HN5 – GB20 – GB8 Bilateral application	Change in MMDs and migraine attacks per 4 weeks – Proportion of patients with a reduction of MMDs by at least 50% during weeks 17 to 20 – VAS – Changes in MSQOL, Pittsburgh Sleep Quality Index (PSQI), MIDAS, Beck Anxiety Inventory (BAI), BDI‐II, and the mean dose of rescue medication from baseline to week 20	Significantly greater reduction in MMDs and migraine attacks per 4 weeks in acupuncture groups as compared to the usual care group during weeks 1 to 20 (acupuncture vs. usual care: −2.4 [−3.5 to −1.4]; *p* < 0.001, 95% CI) Significantly greater reduction in migraine attacks in the sham acupuncture group as compared to usual care (Sham vs. usual care: −0.8 [−1.4 to −0.2]; *p* = 0.008, 95% CI) Significantly higher responder rates and VAS reduction (−2.2 ± 2.5 vs. − 0.9 ± 1.9, *p* < 0.001) in the acupuncture group as compared to the other groups No significant differences in the mean dose of rescue medications or in the BAI and BDI‐II scores across the three groups	Five patients (8%) in the manual acupuncture group reported at least one related adverse effect, whereas no adverse effects were reported in the sham group; however, none of these effects were severe
Yang et al. (2011)	Prospective, randomized, controlled trial, China	Acupuncture group *n* = 33, mean age 47.6 ± 7.4 years, 90% female, 21.3 ± 1.6 migraine days per month, 20.2 ± 1.5 severe headache days per month Topiramate group *n* = 33, mean age 48.1 ± 6.4 years, 86.2% female, 21.0 ± 1.4 migraine days per month, 19.8 ± 1.7 severe headache days per month.	Chronic migraine	– Bilateral BL‐2 – GB‐20 – EX‐HN‐5 – EX‐HN‐3	– MMDs with moderate or severe headache – Changes in MIDAS, HADS, SF‐36, and BDI‐II scores – Intake of acute medications 12 weeks	Greater reduction in MMDs with moderate or severe headache in the acupuncture group as compared to topiramate group 20.2 ± 1.5 days to 9.8 ± 2.8 days vs. Topiramate 19.8 ± 1.7 days to 12.0 ± 4.1 days (*p* < 0.01) Greater improvements in disability, distress, and health‐related quality of life in the acupuncture group as compared to the topiramate group (MIDAS, *p* < 0.01; HADS, *p* < 0.01; BDI‐II, *p* = 0.025; SF‐36, *p* < 0.05) Significantly reduction of acute headache medication intake in both groups with a a significantly greater reduction in the acupuncture group (−9.6 ± 3.3 acupuncture vs. −5.4 ± 4.7 Topiramate; *p* < 0.01)	Fewer patients with side effects in the acupuncture group (6%) as compared to the topiramate group (66%) Acupuncture side‐ effects: local pain, ecchymosis, and local paraesthesia Topiramate side effects: paresthesia (*n* = 16, 48.4%), difficulty with memory (*n* = 12, 36.3%), dyspepsia (*n* = 12, 36.3%), fatigue (*n* = 8, 24.2%), dizziness (*n* = 7, 21.2%), somnolence (*n* = 6, 18.1%), and nausea (*n* = 5, 12.1%)
Yang et al. (2018)	Prospective, randomized, controlled trial, China	Acupuncture group *n* = 21, mean age 34.5 ± 3.3, 6.9 ± 1.5 migraine days per month Flunarizine group *n* = 21, mean age 31.4 ± 2.8 years, 6.37 ± 1.38 migraine days per month	Migraine with or without aura	– Shuai Gu, – Tou Wei – Tai Yang – Feng Chi	– VAS – MMDs – T1: 12 weeks – T2: 24 weeks	Although the VAS showed a significantly greater reduction at T1, no significant differences emerged between the two groups at T3. (*p* > 0.05) MMDs reported same results, at T1 Acupuncture: 3.15 ± 1.27 vs. Flunarizine: 4.53 ± 1.92 (*p* < 0.05). T2: Acupuncture: 4.80 ± 1.88 vs. Flunarizine: 4.47 ± 1.50 (*p* > 0.05)	In the Acupuncture group seven patients (35%) reported Mild subcutaneous hemorrhages Nine patients in the Flunarizine group reported adverse effects: Drowsiness (3 people), constipation (2 people), depression (1 person), and myalgia (1 person)
Zeng et al. (2015)	Prospective, randomized, controlled trial, China	Acupuncture group *n* = 34, mean age 39.9 ± 12.7, 3.2 ± 1.4 migraine days per week Flunarizine group *n* = 34, mean age 40.1 ± 12.2 years, 3.3 ± 1.2 migraine days per week	Migraine with or without aura	– ST8 – ST44 – LI4 – LI11 – LIV3 – SP6 – GB1 – GB14 – GB20 – GV14 – GV20 – EX‐HN3 – EX‐HN5 – HT7	– VAS – SF‐36 – T1: 7 days – T2: 14 days – T3: 21 days	No significant differences between two groups at T1. At T3 At T3, a significant difference is shown in both the VAS (Acupuncture: 1.8 ± 0.2 vs. Flunarizine: 4.03 ± 1.4; *p* < 0.05) and the SF‐36 (Acupuncture:76.6 ± 8.4 vs. Flunarizine: 61.4 ± 6.08; *p* < 0.05)	No reported adverse events
Zhao et al. (2018)	Prospective randomized controlled trial, China	Acupuncture group: *n* = 18, mean age 35.6 ± 4.9 years, 77% female, headache duration 142.1 ± 10.9 h on average, mean monthly days with severe headache 12.5 ± 3.6 Flunarizine group: *n* = 18, mean age 36.6 ± 7.6 years, 72% female, headache duration 149.3 ± 10.5 h on average, mean monthly days with severe headache 11.8 ± 2.7	Chronic migraine	– TE 23 – GB 4 – GB 12 – BL 2 Bilateral application	– MMDs – Headache duration 4 weeks	Significant differences between the two groups in the number of MMDs (acupuncture: 7.2 ± 1.6 vs. Flunarizine: 8.9 ± 1.4, *p* < 0.05) and in the total duration of headache (acupuncture: 92.1 ± 7.2 vs. Flunarizine: 116.1 ± 8.2, *p* < 0.05) at the 4‐weeks follow‐up period	No reported adverse events

### 3.2 Quality Control of Included Studies

**FIGURE 2:**
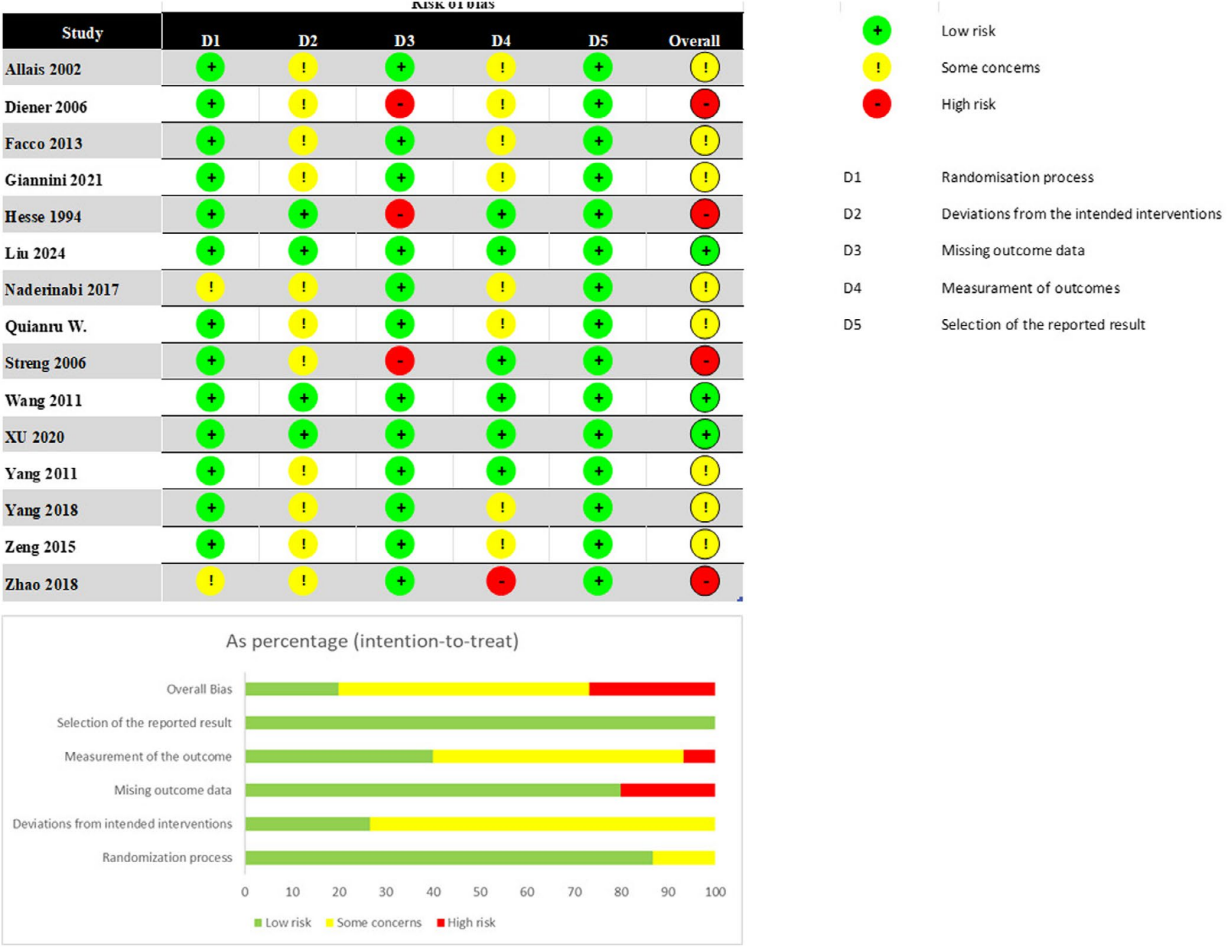
Quality control of included studies.

The summary of the risk of bias among the included RCTs, assessed using the Cochrane RoB 2 tool, is shown in Figure 2. Most studies did not indicate a significant risk of bias in the randomization process. However, two studies [10, 11] raised some concerns due to insufficient information regarding the randomization method and the concealment of patient allocation to intervention groups. 11 out of the 15 studies raised concerns about deviations from the intended interventions, as their open‐label design introduced a potential risk of performance and detection bias [10, 11, 12, 13, 14, 15, 16, 17, 18, 19, 20]. In contrast, the remaining studies utilized double‐blind, double‐dummy protocols, which were consequently assessed as having a low risk of bias [21, 22, 23, 24]. Three studies were evaluated as having a high risk of missing data, attributed to a substantial number of participant dropouts and the lack of an intention‐to‐treat analysis [13, 16, 21]. Eight studies raised some concerns regarding outcome measurement [10, 12, 13, 14, 15, 18, 19, 20]. In these studies, the outcome assessors were aware of the interventions received by participants, which could have influenced the assessment of outcomes. Additionally, the study by Zhao et al. [14] failed to provide information on this aspect, leading to its classification as having a high risk of bias. Conversely, all eligible studies were deemed to have a low risk of bias in the selection of reported results.

### 3.3 Qualitative Analysis

The PICO framework was used to describe the results of individual studies. The study design, clinical characteristics of the sample included, acupoints used, and key results regarding outcomes and adverse events are further summarized in the table.

Allais et al. [12] conducted an RCT involving 160 women with migraine without aura (*Population*) who had not received prophylactic therapy for at least 2 months. The study compared traditional acupuncture (*Intervention* group, *n* = 80) with flunarizine 10 mg (*Comparison* group F, *n* = 80) as prophylactic treatments. Acupuncture was administered weekly for 2 months and then monthly for 4 months, while flunarizine was taken daily for 2 months and then 20 days per month for 4 months. *Outcomes* included monthly migraine attacks, pain intensity, and analgesic intake, assessed at 2, 4, and 6 months. Both groups showed significant reductions in attacks and analgesic use, but pain intensity improved only with acupuncture. Flunarizine led to a faster reduction in attacks after 2 and 4 months, yet a higher percentage of patients in the acupuncture group became pain‐free (12.9% vs. 9.5%). Acupuncture had fewer side effects (sedation: 10%, local pain: 8%) compared to flunarizine (drowsiness: 35%, weight gain: 22%, depression: 7%).

Diener et al. [13] conducted a randomized, double‐blind, controlled trial with 794 migraine patients with or without aura (*Population*) who had not received prophylactic treatment in the past 6 months. The study compared verum acupuncture (*Intervention*), sham acupuncture, and standard prophylactic treatments (beta blockers, flunarizine, or valproic acid) in a 1:1:1 ratio (*Comparison*). Verum and sham acupuncture consisted of 10 30‐min sessions over 6 weeks. Sham acupuncture targeted non‐acupuncture points. The primary *outcome* was the change in migraine days from 4 weeks pre‐randomization to weeks 23–26 post‐randomization. Secondary outcomes included pain intensity, medication use, and quality of life. Migraine days decreased by 2.3 days in the verum group (95% CI 1.9–2.7), 1.5 days in the sham group (95% CI 1.1–2.0), and 2.1 days in the standard therapy group (95% CI 1.5–2.7), with all reductions being significant (*p* < 0.001). No significant differences were found between the groups (*p* = 0.09). Secondary outcomes showed no group differences.

Facco et al. [14] conducted an RCT with 100 migraine patients without aura (*Population*) who had not received prophylactic therapy in the past 6 months. The study compared traditional acupuncture (*Intervention* group, *n* = 50) with valproic acid (*Comparison* group, *n* = 50) for prophylaxis. Acupuncture consisted of two sessions of 10 applications each, administered twice weekly with a 1‐week rest between sessions. *Outcomes* included changes in MIDAS and Pain Relief Scores (PRS) at 3 and 6 months, pain intensity (0–10 scale), rizatriptan use, and treatment side effects. Both groups showed significant reductions in MIDAS, PRS, pain intensity, and rizatriptan use at all time points. Side effects were reported only in the valproic acid group (e.g., nausea, constipation, drowsiness, and weight gain), while no adverse effects occurred in the valproic acid group.

Giannini et al. [15] conducted a randomized, sham‐controlled trial involving 135 migraine patients with or without aura (*Population*) free of prophylactic therapy for at least 3 months. The study compared traditional acupuncture (*Intervention* group, *n* = 69) with standard oral prophylactic treatments (*Comparison* group, *n* = 66, including amitriptyline, beta‐blockers, flunarizine, topiramate, and others). Acupuncture involved 12 sessions over 10 weeks, with semi‐standardized treatments using specific points. The primary *outcome* was the reduction in migraine days between 1 and 4 months as recorded in headache diaries. Secondary outcomes included the proportion of responders (≥ 50% reduction in headache days), migraine attacks, rescue medication use, trial discontinuations, and follow‐up data. Both groups showed significant reductions in headache days, attacks, and medication use, with no differences between groups. Responders were 34.78% in the acupuncture group and 33.33% in the standard therapy group (*p* = 0.477). Mild and reversible side effects were reported with pharmacological treatments, with two cases requiring discontinuation (depression with flunarizine, hypertransaminasemia with topiramate). No adverse events occurred in the acupuncture group.

Hesse et al. [21] conducted an RCT with 77 migraine patients with or without aura (*Population*) who were not receiving prophylactic therapy. The study compared traditional acupuncture (*Intervention* group, *n* = 38) with metoprolol 100 mg daily (*Comparison* group, *n* = 39). Acupuncture sessions, including the number of trigger points, frequency, and total treatments, were tailored to each patient's response, while metoprolol was administered daily for 17 weeks, followed by a 10‐day tapering period, with sham acupuncture mimicking active treatments. *Outcomes* included changes in attack frequency and a global migraine rating (1–3) based on pain severity, duration, and associated symptoms. After 17 weeks, both groups showed significant reductions in attack frequency, with no difference between groups (*p* > 0.20). However, metoprolol was superior for the global rating of attacks (*p* < 0.05). Side effects were more common in the metoprolol group (36%, including fatigue, gastrointestinal symptoms, and dizziness) compared to the acupuncture group (8%, primarily gastrointestinal symptoms).

Liu et al. [22] conducted an RCT with 60 chronic migraine patients (*Population*) who were free of prophylactic therapy for at least 3 months. The study compared traditional acupuncture (*Intervention* group, *n* = 30) with topiramate 50–100 mg/day (*Comparison* group, *n* = 30). Acupuncture was administered in 36 sessions over 12 weeks (three sessions per week), while sham acupuncture and placebo tablets mirrored the active treatments. Primary *outcomes* included changes in monthly migraine days (MMDs) over 12 weeks, along with disability (HIT‐6, MIDAS), quality of life (MSQOL), and psychological profile (BDI‐II, STAI‐T) at week 12. Acupuncture reduced MMDs significantly more than topiramate (−2.79 [95% CI: −4.65 to −0.94, *p* = 0.004] for weeks 1–12; −3.25 [95% CI: −5.57 to −0.92, *p* = 0.007] for weeks 13–24). Acupuncture also led to greater reductions in HIT‐6 and MIDAS scores and to improved quality‐of‐life measures. Adverse events were less frequent with acupuncture (10.0% vs. 26.7%, *p* < 0.001). Acupuncture‐related events included hematoma, numbness, and needling pain, while topiramate caused paresthesia, dizziness, fatigue, nausea, and concentration difficulties.

Naderinabi et al. [10] conducted an RCT on 150 chronic migraine patients (*Population*) free of prophylactic therapy for at least 3 months and without medication overuse headache. They compared traditional acupuncture (*Intervention* group) with botulinum toxin A or sodium valproate 500 mg/day (*Comparison* groups) as prophylactic treatments. Participants were randomly assigned to each group, with 50 patients in each. Acupuncture consisted of 30 sessions over 60 days; botulinum toxin A was injected according to the PREEMPTI protocol, and sodium valproate was taken daily for 3 months. *Outcomes* included headache days, pain severity, medication use, work/school absences, and adverse effects. All treatments significantly reduced headache days and pain severity (*p* = 0.0001), with the acupuncture group showing the best results at all time points. The acupuncture group also had the greatest reduction in medication use (*p* = 0.0001) and missed work or social activities (*p* = 0.0001). Although no significant differences were observed between groups at baseline, T2, or T3, the acupuncture group showed superior results at T1 (*p* = 0.023). The frequency of side effects was significantly lower in the acupuncture group (*p* = 0.021), with acupuncture side effects being mild, such as bleeding or hematoma. In contrast, botulinum toxin A caused ptosis and facial asymmetry, while sodium valproate resulted in side effects like asthenia, weight gain, tremor, and other symptoms.

Qianru et al. [18] conducted a prospective RCT involving 45 migraine patients with or without aura (*Population*). The study compared traditional acupuncture (*Intervention* group, *n* = 22) with flunarizine combined with gabapentin (*Comparison* group F, *n* = 23) as prophylactic treatments. *Outcomes* included monthly migraine attacks, pain intensity, and duration, assessed at 1 and 3 months. Both groups showed significant improvements in outcomes, with the flunarizine/gabapentin group achieving better results. Patients undergoing acupuncture experienced no side effects, whereas those receiving flunarizine/gabapentin reported mild and transient adverse events (dizziness, nausea, and drowsiness).

Streng et al. [16] conducted a prospective RCT involving 114 migraine patients with and without aura (*Population*), free of prophylactic therapy for at least 1 month. The study compared traditional acupuncture (*Intervention* group) with Metoprolol 100–200 mg/day for 12 weeks (*Comparison* group) as prophylactic treatments. Patients were randomly assigned to either acupuncture (*n* = 59) or Metoprolol (*n* = 55). Acupuncture treatment involved 8 to 15 sessions over 12 weeks, while Metoprolol was administered daily for 12 weeks and tapered over 4 weeks. *Outcomes* included changes in migraine days, pain intensity, and disability (SF‐36). The number of migraine days decreased by 2.5 ± 2.9 days in the acupuncture group and 2.2 ± 2.7 days in the Metoprolol group, with no significant difference (*p* = 0.721). Both groups showed reduced pain intensity (*p* = 0.032) and improvement in physical health (*p* = 0.034). However, there were no significant changes in mental health scores (*p* = 0.979). Acupuncture patients reported better results in several pain questionnaire parameters. No serious adverse events were reported in the acupuncture group, whereas six patients in the Metoprolol group experienced severe side effects. All acupuncture patients completed the study, while seven patients in the Metoprolol group dropped out.

Wang et al. [23] conducted a prospective RCT with 140 patients with migraine without aura (*Population*), free from prophylactic therapy for the past 3 months. The study compared traditional acupuncture (*Intervention* group) with flunarizine 5–10 mg/day (*Comparison* group) as a prophylactic treatment. Patients were randomly assigned to either acupuncture plus placebo tablets (*n* = 70) or flunarizine plus sham acupuncture (*n* = 70). The acupuncture group received three 30‐min sessions per week for 4 weeks, while the control group received flunarizine along with three 30‐min sham acupuncture sessions per week. *Outcomes* were assessed at weeks 4 and 16 and included the proportion of patients with a ≥ 50% reduction in migraine days, the change in migraine days, VAS scores, and SF‐36 physical and mental component scores. The acupuncture group had a higher responder rate (41% vs. 28% at week 4, and 39% vs. 26% at week 16, *p* < 0.05) and a greater reduction in migraine days (4.1 ± 3.5 vs. 1.9 ± 2.3 at week 4, and 4.1 ± 3.5 vs. 2.0 ± 2.7 at week 16, *p* < 0.05). However, there were no significant differences in VAS scores or SF‐36 physical and mental health scores (*p* > 0.05). Mild adverse effects were reported in both groups, with five acupuncture patients experiencing minor issues (e.g., mild bleeding, scalp discomfort, and fatigue) and seven control patients experiencing fatigue, faintness, or weight gain.

Xu et al. [24] conducted a randomized controlled clinical trial with a target *population* represented by 150 patients with migraine without aura free of additional prophylactic therapy. The *intervention* investigated was traditional acupuncture plus usual care (*n* = 60). A c*omparison* was made with sham acupuncture plus usual care (*n* = 60) or usual care alone (*n* = 30). All patients received 20 sessions of 30‐min acupuncture treatments or usual care over 8 weeks. *Outcomes* assessed included changes in migraine days, attacks, severity, sleep quality, and other health measures. Results showed that manual acupuncture significantly reduced migraine days and attacks compared to usual care (*p* < 0.001) and reduced attacks more than sham acupuncture (*p* < 0.001). Sham acupuncture also outperformed usual care in reducing migraine attacks (*p* = 0.008). Manual acupuncture showed significant improvements in several outcomes, including VAS, MSQ, PSQI, and MIDAS (*p* < 0.001). However, no significant differences were found between manual and sham acupuncture for most other outcomes, including rescue medication use and anxiety/depression scores (BAI, BDI‐II). Adverse effects were minimal, with only 8% of the manual acupuncture group reporting mild side effects.

Yang et al. [17] conducted a prospective RCT with a target *population* represented by 66 patients with chronic migraine free of prophylactic therapy in the last 3 months. The *intervention* investigated was traditional acupuncture. A *comparison* was made with Topiramate initiated at 25 mg/day as prophylactic treatment. Patients were randomly assigned to the acupuncture group (*n* = 33) or to the Topiramate group (*n* = 33). The acupuncture group received 24 sessions of 30 min of manual bilateral acupuncture over 12 weeks. The control group was treated with Topiramate for 12 consecutive weeks (25 mg/day for 1 week, weekly increases of 25 mg for 4 weeks, and 50 mg/day for 8 weeks). The mean final Topiramate maintenance dose was 84.0 mg/day.

*Outcomes* assessed at baseline and post‐treatment included the monthly number of severe headache days, total headache days, and changes in MIDAS, HADS, SF‐36, and BDI‐II scores, as well as acute medication use. Acupuncture was significantly more effective than Topiramate in reducing severe migraine days (−10.5 ± 2.8 vs. −7.8 ± 3.6; *p* < 0.01). The acupuncture group also showed greater improvements in disability, distress, and quality of life (MIDAS, *p* < 0.01; HADS, *p* < 0.01; BDI‐II, *p* = 0.025; SF‐36, *p* < 0.05). Both groups had reduced acute medication use, but the reduction was more significant in the acupuncture group (−9.6 ± 3.3 vs. −5.4 ± 4.7; *p* < 0.01). In the acupuncture group, 6% of patients reported mild adverse effects like local pain, bruising, and paraesthesia. In contrast, 66% of Topiramate users reported side effects, with the most common being paresthesia (48.4%), memory issues (36.3%), dyspepsia (36.3%), fatigue (24.2%), dizziness (21.2%), somnolence (18.1%), and nausea (12.1%).

Yang et al. [19] conducted an RCT involving 42 patients with migraine with or without aura (Population), with a history of migraine for at least 1 year, age at onset < 45 years, and the presence of at least two attacks per month in the last 3 months. Patients who had received preventive treatments for migraine or acupuncture in the last 6 months were excluded. The study compared traditional acupuncture (Intervention group, *n* = 21) with flunarizine (Comparison group F, *n* = 21) as prophylactic treatments. Acupuncture was administered 2–3 sessions per week, each lasting 20 min, for 4 weeks, while flunarizine was taken daily for 2 months, starting with a dosage of 5 mg for 2 weeks and then increased to 10 mg. Outcomes included the headache index, calculated by multiplying the pain score (based on VAS assessment) by the duration score (classified into five levels), summing the attacks over a unit of time. Effectiveness was expressed as the percentage reduction in the headache index compared to baseline. A reduction of ≥ 90% indicated clinical recovery, 55%–89% significant improvement, 20%–54% moderate improvement, and < 20% ineffective therapy. Both the acupuncture group and the pharmacological group showed a preventive effect on migraines, significantly reducing pain intensity, headache days, and attack frequency. After 4 and 12 weeks of treatment, the effectiveness of the acupuncture group was superior to that of the pharmacological group, but after 24 weeks, no significant differences were found between the two groups. In the acupuncture group, seven individuals experienced mild subcutaneous hemorrhages without bruising, and no participants dropped out due to adverse events. In the pharmacological group, nine individuals experienced adverse effects, with seven requiring a dose reduction. These effects included drowsiness (three individuals), constipation (two individuals), depression (one individual), and myalgia (one individual). Two participants discontinued the study due to intolerance to the side effects (drowsiness).

Zeng et al. [20] conducted a randomized controlled clinical trial with a target population represented by 68 patients with migraine free of additional prophylactic therapy. The intervention investigated was traditional acupuncture (*n* = 34). A comparison was made with flunarizine 5 mg (*n* = 34). Both treatments were administered for 3 weeks. The outcomes included the number of migraine attacks per week, the average duration of attacks, pain intensity assessed with the VAS scale, matrix metalloproteinase‐2 (MMP‐2) activity in serum, and quality of life measured with the SF‐36 questionnaire. Results showed that before treatment and after 7 days, there were no significant differences between the groups in terms of attack frequency, average headache duration, VAS, and MMP‐2 activity (*p* > 0.05). However, after 14 and 21 days of treatment, the acupuncture group showed a reduction in frequency, duration, and intensity of attacks together with a reduced serum MMP‐2 activity (*p* < 0.05). Additionally, in the acupuncture group, a positive correlation was found between the reduction in VAS scores and the decrease in serum MMP‐2 activity. No adverse events were reported.

Zhao et al. [11] conducted an RCT with a target *population* represented by 36 patients with chronic migraine lasting over 1 year, who had not received preventive treatment in the past year. The *intervention* investigated was traditional acupuncture. A *comparison* was made with Flunarizine as prophylactic treatment. Patients were randomized to the acupuncture group (*n* = 18) or the Flunarizine group (*n* = 18). Patients in the acupuncture group underwent three weekly sessions of traditional acupuncture for 4 weeks, with needles retained at the acupoints for 24 h per session. Patients in the control group received Flunarizine hydrochloride capsules, 10 mg per day, for 4 weeks. Headache severity and duration were recorded for 1 month at the end of the treatment, while headache days per month and total headache duration were tracked during the 4‐week follow‐up. *Outcomes* at the 4‐week follow‐up showed statistically significant differences between the two groups in the number of headache days (acupuncture: 7.2 ± 1.6 vs. Flunarizine: 8.9 ± 1.4, *p* < 0.05) and in the total duration of headache (acupuncture: 92.1 ± 7.2 vs. Flunarizine: 116.1 ± 8.2, *p* < 0.05). No adverse effects were reported in this study.

**FIGURE 3:**
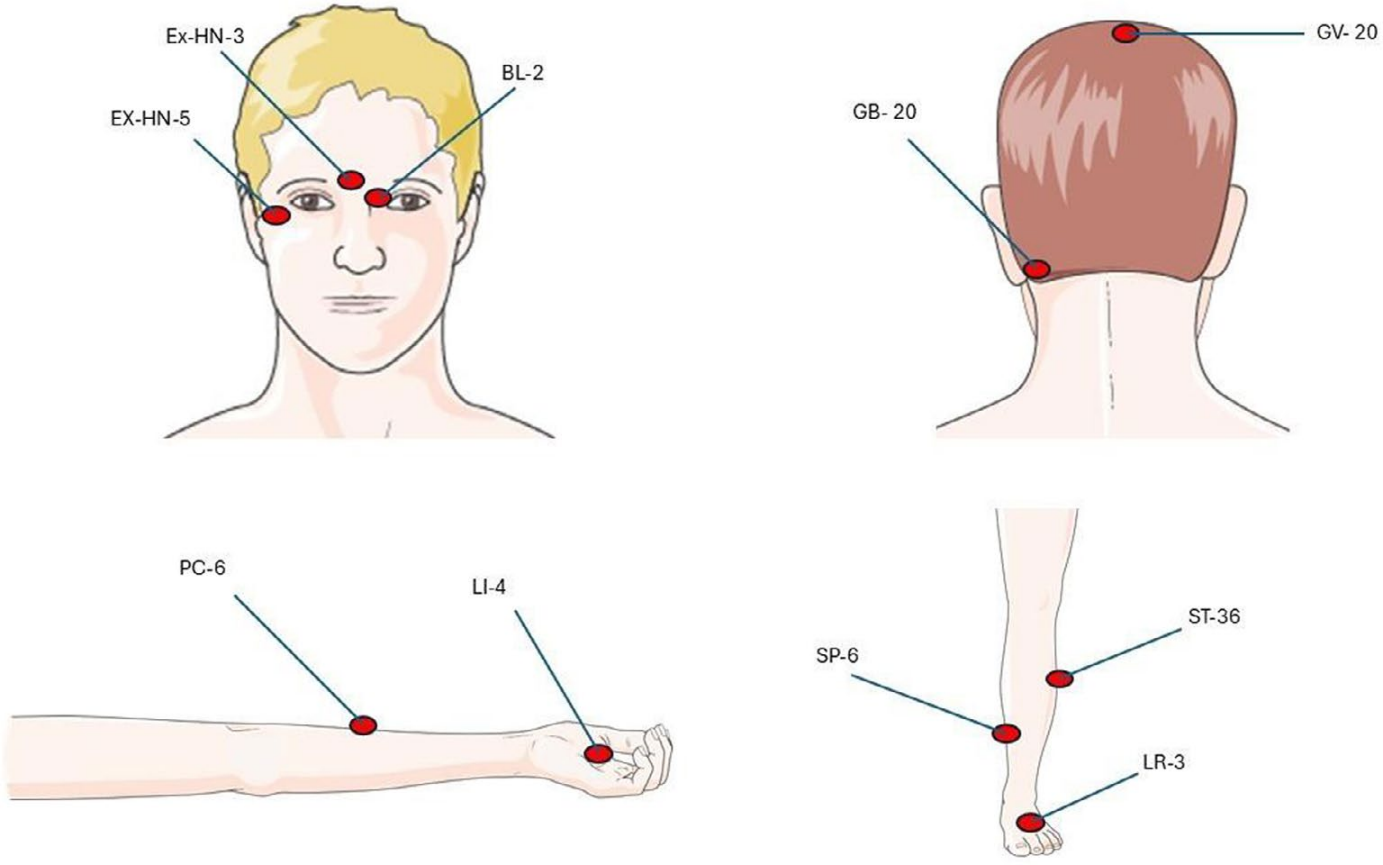
Location of the main acupoints used in the included studies. *Source:* The figure was partly generated using Servier Medical Art, provided by Servier, licensed under a Creative Commons Attribution 3.0 unposted license.

The locations of the main acupoints used in these studies are summarized in Figure 3.

### 3.4 Quantitative Analyses

In all studies, the baseline comparison between samples indicated a condition of homogeneity, with no significant differences observed, either at the demographic or clinical level.

**FIGURE 4:**
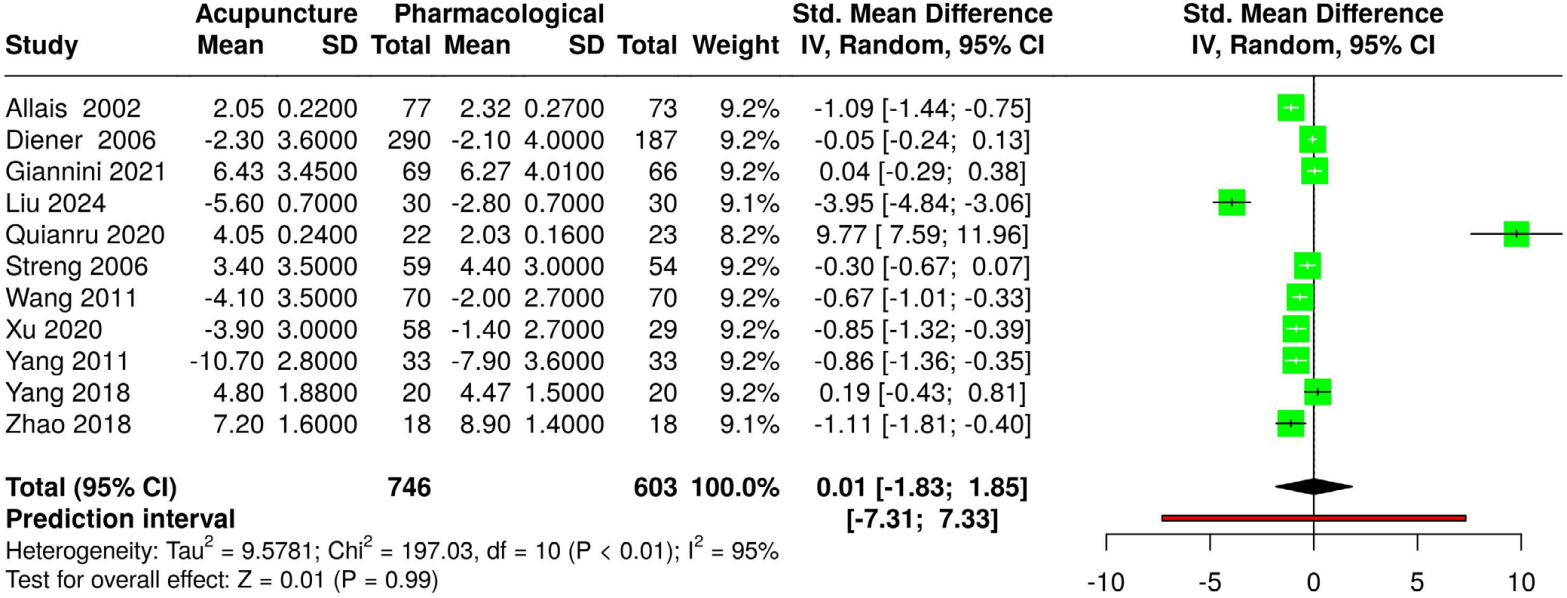
Forest plot of migraine days.

Regarding the number of migraine days per month, the analysis conducted using a random effects model with the inverse variance method to compare the SMD revealed no statistically significant difference between the two cohorts (SMD: 0.01, 95% CI: −1.83 to 1.85, *I* ^2^ = 95%, 11 studies, 1349 participants, moderate quality of evidence). A significant heterogeneity was detected (*p* < 0.01 and *I* ^2^ = 95%); these data indicate that the variability of the studies arises from heterogeneity rather than random chance. Results are shown in Figure 4.

**FIGURE 5:**
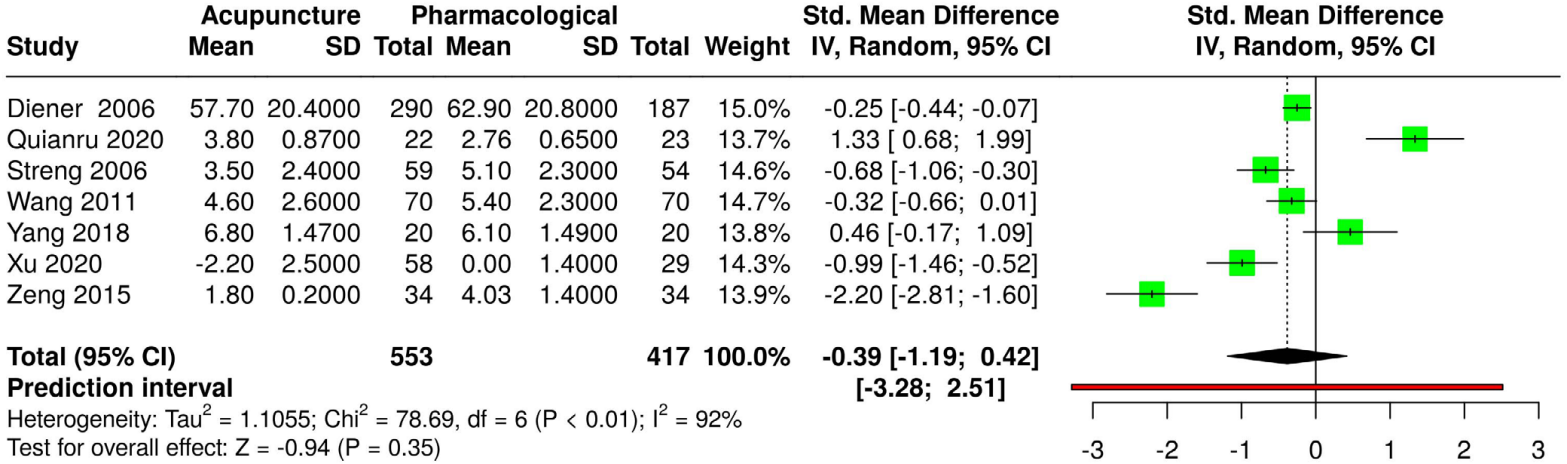
Forest plot of pain intensity.

As well, as shown in Figure 5, no statistically significant difference was observed for pain intensity (SMD: −0.39, 95% CI: −1.19; 0.42, *I* ^2^ = 92%, 7 studies, 970 participants, moderate quality of evidence).

Finally, the funnel plots were considered uninformative because < 10 studies were included in the meta‐analysis of each outcome, so we were unable to inspect the presence of publication bias [8].

## 4 Discussion

The synthesis of available evidence suggests that acupuncture can be added to the therapeutic toolkit for patients with migraine. However, it should be emphasized that acupuncture should not be proposed as a replacement for other pharmacological preventive therapies, which are considered effective and often decisive for patients. Nevertheless, the effectiveness and safety profile of acupuncture should still be highlighted, and it can be offered as an adjunctive therapy for patients or as an alternative treatment when conventional therapies cannot be used.

This systematic review and meta‐analysis, as compared to others, includes only studies comparing acupuncture and drugs in the prophylactic management of migraine, without considering the role of acupuncture as an acute treatment for migraine. Given the extreme heterogeneity of the studies in this field, with outcomes that are highly variable and difficult to compare, separating the treatment of the acute phase from the preventive phase is essential to provide clarity. Furthermore, we used a search string that included all pharmacological therapies currently used for prevention and authorized by the guidelines, including CGRP‐targeting pharmacological therapies, which have not been considered as comparators so far. Findings suggest that the effects of acupuncture and its tolerability are sustained over time, indicating that it can be used long‐term by patients for both episodic and chronic forms.

Certainly, these conclusions warrant some reflections and considerations: first, comparative trials on acupuncture refer exclusively to traditional preventive therapies, while no comparative data are yet available between acupuncture and innovative therapies that act on CGRP ligand or its receptor. These therapies, including monoclonal antibodies and gepants, demonstrate high efficacy and tolerability, although their use is not generalizable to all migraine patients due to drug reimbursement issues, with significant variations across countries. Nonetheless, the high selectivity of these drugs makes them the first choice according to the latest guidelines [25], indicating that the efficacy of acupuncture, either as an alternative or adjunctive therapy, should also be evaluated in comparison to these new medications. Another consideration regarding the efficacy of acupuncture as a prophylactic treatment arises from the extreme heterogeneity of the reported studies: although most studies show a rigorous patient randomization, there are some factors that may have introduced bias into the results. In some studies, the dropout rate is high and may have compromised the validity of the results. Dropout in RCTs can affect the outcomes, especially when there are differing dropout rates between treatment groups, leading to fewer patients being followed up in one arm compared to the other [26]. This is evident in the study by Streng et al. [16], where the high dropout rate in the metoprolol group may have influenced the results. The authors attribute this high dropout rate to a more positive attitude toward acupuncture compared to metoprolol, as well as the differing adverse effect profiles between the treatment groups. Although unequal dropout rates between treatment groups in a clinical trial do not always introduce bias, caution is needed when interpreting the results [26]. Another factor that may influence the comparability between studies and the generalizability of the results to all migraine sufferers is the heterogeneity of acupuncture protocols used: the heterogeneity involves different aspects, such as the selection of acupuncture points, the overall duration of treatment, the frequency of sessions, and the duration of individual treatments. Moreover, the technique used by each acupuncturist, as well as their level of training and expertise, can introduce a potential source of bias. Another issue that warrants further discussion is the sham‐controlled design used in some studies; successful patient blinding in sham acupuncture is difficult to achieve and this can influence the validity and reliability of study results [27]. The sham procedure involves the use of needles that do not penetrate the skin or stimulate areas not considered sensitive in acupuncture, thus not exerting specific therapeutic effects. The ideal sham acupuncture device should mimic all aspects of real acupuncture but be inert. However, true inert acupuncture is not credible, as the stimulation of any point, even those distant from conventionally recognized acupuncture points, is still expected to generate some effect, regardless of its therapeutic benefits. Moreover, even when patients are blinded to the treatment, the acupuncturist remains unblinded, making it very challenging to conduct a true double‐blind trial. Another point of discussion is the placebo effect associated with acupuncture, which may partly explain its efficacy in the treatment of pain. The presence of a placebo component is supported by evidence suggesting a minimal effect associated with sham stimulation: minimal acupuncture control conditions, where non‐acupuncture points are needled, true points are gently stimulated, or placebo needles that do not penetrate the skin are used, are associated with a small but yet detectable effect [28, 29]. Variations in the effect of placebo can be partly explained by variations in how trials were conducted and how patients are informed [29]. Certainly, sham acupuncture conditions used as placebo interventions cannot be considered equivalent to oral pharmacological placebos, as they are not entirely inert and exert an effect, albeit minimal, on nociceptive processes. In this respect, a recent network meta‐analysis confirmed that sham acupuncture and sham surgery are associated with higher responder ratios than oral pharmacological placebos [30]. Part of the enhanced placebo effect may stem from increased expectations for the treatment, the physical interaction between the clinician and the patient, and from the physiological effects of skin stimulation. Therefore, clinicians who treat patients with migraine should be aware that the method of treatment delivery might have an important influence on outcome [30]. These observations make the comparison between true acupuncture and other infiltrative techniques, such as subcutaneous injection of botulinum toxin type A, particularly interesting [13], in this regard, comparisons between acupuncture and anti‐CGRP monoclonal antibodies administered subcutaneously (erenumab, fremanezumab, galganezumab) or intravenously (eptinezumab) will be equally intriguing. Finally, differences between personalized and formula acupuncture should be considered.

Regardless of these considerations, acupuncture could be highly beneficial for some subgroups of patients, including adolescents, pregnant women, and individuals with a long history of chronic migraine. The former can benefit from acupuncture due to its excellent tolerability and the low incidence of side effects [31, 32], while the latter may benefit from the acupuncture‐related reversal of central sensitization processes [33].

In conclusion, the use of acupuncture can help expand our therapeutic toolkit available for patient care, especially for the more vulnerable individuals and those with more complex conditions. Future evidence will show how acupuncture treatment may be integrated with the latest and most selective treatments.

## Author Contributions

**Francesca Pistoia:** conceptualization, data curation, formal analysis, investigation, methodology, supervision, writing – original draft, writing – review and editing. **Simone Cesarano:** data curation, formal analysis, methodology, writing – original draft, writing – review and editing. **Gennaro Saporito:** data curation, formal analysis, investigation, methodology, writing – review and editing. **Maria Albanese:** methodology, investigation, writing – review and editing. **Cecilia Lucenti:** methodology, writing – review and editing. **Secondo Scarsella:** methodology, writing – review and editing. **Aldo Liguori:** methodology, investigation, writing – review and editing. **Federica Umani Ronchi:** conceptualization, methodology, investigation, writing – original draft, writing – review and editing.

## Conflicts of Interest

The authors declare no conflicts of interest.

## Data Availability

The data that support the findings of this study are available from the corresponding author upon reasonable request.
